# College and Hospital Notes

**Published:** 1904-12

**Authors:** 


### COLLEGE AND HOSPITAL NOTES.

The University of Buffalo, with a view to the ultimate formation of a department of liberal arts, has arranged for a series of lectures, by accomplished scholars, to be given under the auspices of the university. These courses are for instruction and not for entertainment. They are primarily planned for the benefit of teachers and students, who, it is believed, will welcome such an opportunity for self-culture and training.

The lectures will be given in the new and commodious classrooms on the fourth floor of the Y. M. C. A. Building, corner of Mohawk and Genesee streets. The association is heartily in sympathy with the university and has courteously offered the use of its rooms for this purpose. Detailed information regarding the work may be had by addressing Lecture-study Department, University of Buffalo, Y. M. C. A. Building, Buffalo, N. Y.

---

The New York Post-Graduate Hospital and Medical School recently became the recipient of a generous gift from an unknown source, which has enabled it to open an annex to its dispensary for the treatment of pulmonary tuberculosis. Gratified at the way his friend had been cured of incipient tuberculosis at the dispensary, the anonymous giver has leased a boarding house at No. 322 East Nineteenth street, and at a cost of several thousand dollars overhauled, refitted and completely converted it, and has also promised $6,000 yearly for its maintenance for a certain term of years. The annex has twelve beds, and for the first time in the history of the hospital and medical school, poor patients in the advanced stages of pulmonary tuberculosis will be able to receive constant individual treatment and attention until the crisis of their cases shall be past.

As indicating what may be done under systematic management, an independent board of physicians has already decided that out of some hundred and fifty cases of pulmonary tuberculosis in its incipient stages, treated at the dispensary in six or seven years, more than forty have resulted in complete recoveries. Surely, this anonymous friend of the post-graduate tuberculosis annex has performed a philanthropic act that puts all of Mr. Carnegie's library gifts far in the shade!!

---

The Hackley Hospital, Muskegon, Mich., was dedicated November 17, 1904. Mr. Charles H. Hackley made a gift to it of $220,000, having already endowed the institution with $100,000 in 6 per cent. bonds, and also donated $40,000 for the establishment of four free beds.

